# Telehealth Utilization and Associations in the United States During the Third Year of the COVID-19 Pandemic: Population-Based Survey Study in 2022

**DOI:** 10.2196/51279

**Published:** 2024-04-26

**Authors:** Jiyeong Kim, Zhuo Ran Cai, Michael L Chen, Sonia Onyeka, Justin M Ko, Eleni Linos

**Affiliations:** 1 Stanford Center for Digital Health School of Medicine Stanford University Stanford, CA United States

**Keywords:** telehealth, telemedicine, digital health, e-health, e-medicine, utilization, population-based study, clinical practice, healthcare delivery, sociodemographic factor, COVID-19, pandemic

## Abstract

**Background:**

The COVID-19 pandemic rapidly changed the landscape of clinical practice in the United States; telehealth became an essential mode of health care delivery, yet many components of telehealth use remain unknown years after the disease’s emergence.

**Objective:**

We aim to comprehensively assess telehealth use and its associated factors in the United States.

**Methods:**

This cross-sectional study used a nationally representative survey (Health Information National Trends Survey) administered to US adults (≥18 years) from March 2022 through November 2022. To assess telehealth adoption, perceptions of telehealth, satisfaction with telehealth, and the telehealth care purpose, we conducted weighted descriptive analyses. To identify the subpopulations with low adoption of telehealth, we developed a weighted multivariable logistic regression model.

**Results:**

Among a total of 6252 survey participants, 39.3% (2517/6252) reported telehealth use in the past 12 months (video: 1110/6252, 17.8%; audio: 876/6252, 11.6%). The most prominent reason for not using telehealth was due to telehealth providers failing to offer this option (2200/3529, 63%). The most common reason for respondents not using offered telehealth services was a preference for in-person care (527/578, 84.4%). Primary motivations to use telehealth were providers’ recommendations (1716/2517, 72.7%) and convenience (1516/2517, 65.6%), mainly for acute minor illness (600/2397, 29.7%) and chronic condition management (583/2397, 21.4%), yet care purposes differed by age, race/ethnicity, and income. The satisfaction rate was predominately high, with no technical problems (1829/2517, 80.5%), comparable care quality to that of in-person care (1779/2517, 75%), and no privacy concerns (1958/2517, 83.7%). Younger individuals (odd ratios [ORs] 1.48-2.23; 18-64 years vs ≥75 years), women (OR 1.33, 95% CI 1.09-1.61), Hispanic individuals (OR 1.37, 95% CI 1.05-1.80; vs non-Hispanic White), those with more education (OR 1.72, 95% CI 1.03-2.87; at least a college graduate vs less than high school), unemployed individuals (OR 1.25, 95% CI 1.02-1.54), insured individuals (OR 1.83, 95% CI 1.25-2.69), or those with poor general health status (OR 1.66, 95% CI 1.30-2.13) had higher odds of using telehealth.

**Conclusions:**

To our best knowledge, this is among the first studies to examine patient factors around telehealth use, including motivations to use, perceptions of, satisfaction with, and care purpose of telehealth, as well as sociodemographic factors associated with telehealth adoption using a nationally representative survey. The wide array of descriptive findings and identified associations will help providers and health systems understand the factors that drive patients toward or away from telehealth visits as the technology becomes more routinely available across the United States, providing future directions for telehealth use and telehealth research.

## Introduction

Telehealth refers to health care delivered through digital devices (eg, computers, tablets, telephones, or smartphones) and typically includes communicating with the health care providers via live chat over the audio or video format or asynchronous messages through email or a patient portal [1]. During the early COVID-19 pandemic, with the unprecedented “Stay at Home Order” in March 2020 in the United States, in-person office visits were extremely restrained, while the demand of health care significantly increased due to the widespread infectious disease outbreak. Telehealth quickly served as an essential alternative to the limited in-person care [2]. Many private and public health insurance plans promptly started to cover virtual visits to respond to these emergency situations in an effort to enhance the availability of telehealth access [3,4]. The majority of health care sectors, including oncology, psychology, and surgery, promptly provided telehealth services to address the care needs while avoiding unnecessary in-person exposure to the infectious virus [5-7].

Previous studies analyzing sociodemographic characteristics have shown lower telehealth adoption in some populations in the United States. A study using one primary care network reported that older adults, non-Hispanic Whites, and low-income individuals were less likely to utilize telehealth visits [8]. Women and Medicaid beneficiaries had low odds of using telehealth among cancer survivors [9]. Moreover, low English proficiency was also related to low telehealth adoption among New York residents [10]. However, previous assessments were conducted in limited population groups (eg, older African American individuals) [11], at a single medical center or academic institution, with a specific medical specialty area (eg, oncology surgery), or in one geographic area (eg, New York, Los Angeles) and solely examined trends of telehealth utilization [12] or the impact of parity payment laws on telehealth use [13].

However, many components of telehealth use, such as telehealth availability, motivations for using telehealth, and patient perceptions of telehealth visits remain unknown. Second, an analysis of sociodemographic factors associated with low adoption of telehealth among individuals in the United States has not been conducted at the population level. Third, prior studies were conducted in the early phases of the pandemic, which may not be representative of late pandemic and current telehealth practices. Thus, this study aimed to comprehensively assess telehealth use in the United States, including adoption, motivations to and purposes of use, satisfaction, and perceptions, and identify sociodemographic associations with low adoption of telehealth during the third year of the COVID-19 pandemic using a nationwide database. Our findings will advance knowledge of recent telehealth use in the United States and contribute to preparing targeted approaches to enhance telehealth among those with low adoption of it. Recent evidence suggests that telehealth could contribute to enhancing health care access in some marginalized subgroups [14]. Hence, the knowledge and effort will be timely because telehealth has now been grounded as an essential part of health care delivery, being promoted from an emergency alternative during the early COVID-19 pandemic [15].

## Methods

### Data Source

We used a nationally representative survey (Health Information National Trends Survey [HINTS] 6, 2022) for the study [16]. HINTS is a publicly available source of self-reported cross-sectional data. The survey was administered to noninstitutionalized civilians (≥18 years) in the United States who were selected by a random sampling of stratified addresses. HINTS 6 was offered as a paper or online survey and collected from March 2022 through November 2022. With a total of 6252 respondents, the response rate was 28.1% [17]. We applied the full-sample weights to account for the household-level base weight, nonresponse, and the person-level initial weight [17]. This study followed the Strengthening the Reporting of Observational Studies in Epidemiology (STROBE) guidelines [18].

### Outcome

To evaluate telehealth adoption, the following question was used: “A telehealth is a telephone or video appointment with a doctor or health professional. In the past 12 months, did you receive care from a doctor or health professional using telehealth?” The response options were “yes” (received by video, audio, or both) or “no” (not received; see Multimedia Appendix 1).

To assess motivation, those who answered “yes” to the telehealth adoption question were asked to indicate reasons why they chose telehealth, with answer options including (1) the health care provider recommended or required it, (2) I wanted advice about whether I needed in-person medical care, (3) I wanted to avoid possible infection at the office, (4) it was more convenient than going to the doctor, and (5) I could include family or caregivers in my appointment. Answer choices included “yes” or “no.” To examine satisfaction with their telehealth visits, participants used a Likert scale (strongly agree, somewhat agree, somewhat disagree, strongly disagree) to rate the following items: (1) I had technical problems with my telehealth visits, (2) the telehealth care was as good as in-person care, (3) I was concerned about the privacy of my telehealth visits. To assess the purpose of telehealth use, participants also used a Likert scale (strongly agree, somewhat agree, somewhat disagree, strongly disagree) to rate the following items: (1) annual visit, (2) minor illness/acute care, (3) managing chronic condition/disease, (4) medical emergency, (5) mental health, (6) other. For this study, the responses to the latter 2 questions were then coded as a binary variable: agree (strongly agree, somewhat agree) or disagree (somewhat disagree, strongly disagree).

To evaluate their perceptions, those who answered “no” to the telehealth adoption question were asked to indicate the reasons why they did not participate in telehealth visits, answering “yes” or “no” to options including (1) a preference for in-person care, (2) privacy concerns, or (3) difficulty with use.

### Covariates

We selected sociodemographic characteristics based on the social determinants of health conceptual framework from Healthy People 2030 [19], which includes age, birth gender, race/ethnicity, household income (<US $20,000, US $20,000 to <$35,000, US $35,000 to <$50,000, US $50,000 to <$75,000, ≥US $75,000), education, marital status, employment status, health insurance, and rurality of residence (metropolitan, micropolitan, small town, rural) [20,21]. Additionally, health status factors included general health (excellent, very good, good, fair, poor) and chronic medical conditions (cancer, diabetes, high blood pressure, heart disease, lung disease).

### Statistical Analysis

We performed weighted descriptive analyses to calculate the frequency (n) and weighted percentage (%) with the SE to illustrate the sociodemographic and health status characteristics of the study population. Weighted descriptive analyses were also conducted to present telehealth adoption (computed by prevalence), by mode, sociodemographic characteristics, and health status characteristics to identify subgroups with higher-than-average adoption, as well as motivations for use, the care purpose, satisfaction with recent telehealth visits, and perception of telehealth prior to its use. Differences by group were assessed using the Wald chi-square test. To further explore differences in purpose and satisfaction with recent telehealth visits by age, birth gender, race/ethnicity, education, and employment status, weighted descriptive analyses with the Wald chi-square test were also performed. To examine the factors associated with telehealth use, we developed a multivariable weighted logistic regression model to obtain odds ratios (ORs) and 95% CIs for the sociodemographic and health status characteristics related to telehealth use. The logistic regression model was adjusted for age, birth gender, race/ethnicity, education, income, marital status, employment status, health insurance, number of health care office visits a year, and general health status, which were selected because these were potential confounders in this study (eg, changed covariate estimates by more than 10%) or previously known confounders for telehealth use [8,9,11].

The range of missingness was 1.7% to 11.4%, and covariates with any missing values were imputed. We applied the Hot deck imputation method, which was used to account for nonresponse by HINTS [16]. For all the descriptive and regression analyses, the imputed data were used, and the statistical significance was determined at *P*<.05 in SAS 9.4 (SAS Studio) [22,23].

### Ethical Considerations

This was a secondary analysis of publicly available national survey data (HINTS). This study did not involve human subjects or identifiable information. Given that the data were deidentified, this study was deemed exempt from review by the Institutional Review Board of Stanford University.

## Results

### Study Population Characteristics

Table 1 presents the sociodemographic and health status characteristics of the study population in the third year of the COVID-19 pandemic in the United States. Of the survey participants, 78.6% (4045/6252) of the respondents were less than 65 years old, 50.5% (3733/6252) were women, 61% (3615/6252) were non-Hispanic White, 72% (4703/6252) had some or more than a college education, 62.4% (3558/6252) had an income of at least US $50,000, slightly more than one-half were employed (2980/6252, 54.1%), slightly more than one-half were married (3234/6252, 56.1%), 85.7% (5393/6252) resided in a metropolitan area, 89.2% (5709/6252) had health insurance, 83.2% (5134/6252) were in generally excellent or good health, and 37.1% (2798/6252) reported high blood pressure.

**Table 1:** Sociodemographic and health status characteristics of 6252 US adults in the third year of the COVID-19 pandemic (Health Information National Trends Survey [HINTS] 6, 2022).

Characteristics			Respondents, n^a^		Respondents, weighted % (SE)
**Age (years)**					
	18-34	979		26.2 (0.8)	
	35-49	1262		25.1 (0.9)	
	50-64	1804		27.3 (0.6)	
	65-74	1362		12.9 (0.1)	
	≥75	848		8.5 (0.04)	
**Gender**					
	Female	3733		50.5 (0.4)	
	Male	2519		49.5 (0.4)	
**Race/ethnicity**					
	Non-Hispanic White	3615		61.0 (0.4)	
	Non-Hispanic Black/African American	955		10.6 (0.2)	
	Hispanic	1124		17.3 (0.3)	
	Non-Hispanic Asian	343		6.0 (0.2)	
	Others	215		5.1 (0.2)	
**Education**					
	Less than high school	406		6.5 (0.6)	
	High school graduate	1143		21.5 (0.8)	
	Some college	1851		39.7 (0.8)	
	At least a college education	2852		32.3 (0.3)	
**Household income (US $)**					
	<20,000	1064		14.7 (0.9)	
	20,000 to <35,000	814		11.5 (0.7)	
	35,000 to <50,000	816		11.4 (0.7)	
	50,000 to <75,000	1062		18.1 (0.9)	
	≥75,000	2496		44.3 (1.0)	
**Employment^b^**					
	Employed	2980		54.1 (1.3)	
	Unemployed	3272		45.9 (1.3)	
**Marital status^c^**					
	Married	3234		56.1 (0.5)	
	Not married	3018		43.9 (0.5)	
**Rurality^d^**					
	Metropolitan	5393		85.7 (0.7)	
	Micropolitan	489		7.8 (0.5	
	Small town	241		4.3 (0.6)	
	Rural	129		2.2 (0.3)	
**Health insurance^e^**					
	Yes	5709		89. 2 (0.2)	
	No	543		10.8 (0.2)	
**General health status**					
	Excellent/good	5134		83.2 (1.0)	
	Fair/poor	1118		16.8 (1.0)	
**Chronic medical condition**					
	Diabetes	1342		17.1 (0.7)	
	High blood pressure	2798		37.1 (0.9)	
	Heart disease	617		7.6 (0.5)	
	Lung disease	850		11.7 (0.5)	
	Cancer	937		10.2 (0.2)	

^a^Covariates with any missing values were imputed; missingness of covariates from the sample of 6252 adults: age: n=98, 1.6%; birth gender: n=410, 6.6%, race/ethnicity: n=687, 11%; education: n=404, 6.5%; income: n=732, 11.7%; employment status: n=390, 6.2%; marital status: n=415, 6.6%; health insurance: n=126, 2%; general health status: n=234, 3.7%; diabetes: n=252, 4%; high blood pressure: n=244, 3.9%; heart disease: n=238, 3.8%; lung disease: n=234, 3.7%, cancer: n=370, 5.9%.

^b^Employment status (employed vs unemployed including homemaker, student, retired, disabled).

^c^Marital status (married or living with a romantic partner as a married couple vs not married including divorced, widowed, separated, single, or never been married).

^d^HINTS used the Urban-Rural Commuting Area (RUCA), which categorizes census tracts based on population density, urbanization, and commuting patterns developed by the United States Department of Agriculture to determine the rurality of residence of the respondents.

^e^Covered by any kind of health insurance or health care plan, including employer-sponsored insurance; prepaid plans; or government plans such as Medicare, Medicaid, or Tricare.

### Telehealth Adoption by Mode by Sociodemographic Characteristics

Overall, 39.3% (2517/6252) of the respondents had adopted telehealth (Table 2). Video-only visits (17.8%) were more prevalent, followed by audio-only visits (11.6%). Table 2 illustrates telehealth adoption by mode and the differences by sociodemographic and health status characteristics. Video visit adoption was higher among those aged 35 years to 49 years (*P*<.001), women (*P*<.001), the employed (*P*<.001), the insured (*P*<.001), those with at least a college education (*P*<.001), and individuals with a high income (≥US $75,000; *P*<.001). Audio visit adoption was higher among the oldest age group (≥75 years; *P*<.001), Hispanic individuals (*P*=.02), those with a low income (<US $35,000; *P*<.001), and unemployed respondents (*P*<.001).

**Table 2:** Telehealth adoption by sociodemographic and health status characteristics of 6252 US adults in the third year of the COVID-19 pandemic (Health Information National Trends Survey [HINTS] 6, 2022).

Characteristics		Any^a^					Video only, weighted % (SE)		Audio only, weighted % (SE)		Both video and audio, weighted % (SE)		*P* value	
		Participants, n	Participants, weighted % (SE)	*P* value										
All adopters (n=6046)		2517	39.3 (1.0)	—^b^		17.8 (0.8)		11.6 (0.6)		8.4 (0.5)		—		
**Age (years)**					.009									<.001
	18-34 (n=916)	385	35.7 (2.6)			15.8 (2.3)		8.6 (1.5)		9.5 (1.5)^c^				
	35-49 (n=1226)	566^c^	45.1 (2.1)^c^			22.7 (1.7)^c^		12.2 (1.3)^c^		8.4 (1.2)				
	50-64 (n=1755)	728	39.0 (2.1)			18.9 (1.4)^c^		10.7 (0.9)		8.6 (1.2)^c^				
	65-74 (n=1324)	531	37.7 (2.3)			15.5 (1.5)		14.7 (1.4)^c^		6.5 (0.8)				
	≥75 (n=825)	307	36.2 (2.5)			9.6 (1.6)		17.9 (2.2)^c^		7.4 (1.5)				
**Gender**					<.001									<.001
	Female (n=3607)	1613^c^	44.1 (1.5)^c^			19.5 (1.0)^c^		13.2 (0.9)^c^		9.9 (0.8)^c^				
	Male (n=2439)	904	34.4 (1.5)			16.2 (1.2)		10.0 (0.9)		6.9 (1.0)				
**Race/ethnicity**					.37									.02
	Non-Hispanic White (n=3506)	1440	39.1 (1.4)			18.5 (1.1)^c^		11.2 (1.0)		8.2 (0.8)				
	Non-Hispanic Black/African American (n=933)	372	35.4 (3.1)			15.0 (2.3)		10.6 (1.2)		8.0 (1.9)				
	Hispanic (n=1073)	479^c^	39.8 (2.1)^c^			15.4 (1.7)		13.9 (1.2)^c^		8.9 (1.4)^c^				
	Non-Hispanic Asian (n=327)	127	38.5 (5.6)			18.9 (4.1)^c^		11.2 (3.0)		8.0 (2.1)				
	Others (n=207)	99^c^	49.5 (8.2)^c^			23.1 (9.2)^c^		11.8 (3.6)^c^		10.3 (3.2)^c^				
**Education**					<.001									<.001
	Less than high school (n=389)	125	29.8 (4.9)			13.5 (4.3)		11.2 (1.6)		3.2 (1.1)				
	High school graduate (n=1097)	376	33.7 (1.9)			13.4 (1.7)		12.3 (1.6)^c^		6.9 (1.8)				
	Some college (n=1758)	710	39.2 (1.7)			17.5 (1.5)		11.3 (1.0)		8.7 (1.1)^c^				
	At least a college graduate (n=2802)	1306^c^	45.0 (1.6)^c^			22.1 (1.2)^c^		11.7 (0.8)^c^		10.1 (0.9)^c^				
**Household income (US $)**					.10									<.001
	<20,000 (n=1012)	402	36.1 (2.8)			13.6 (1.9)		12.9 (1.3)^c^		7.8 (1.7)				
	20,000 to <35,000 (n=775)	281	36.2 (2.7)			12.7 (2.7)		14.8 (2.0)^c^		6.8 (1.5)				
	35,000 to <50,000 (n=772)	311	37.9 (3.5)			15.9 (2.2)		10.9 (1.3)		10.0 (2.7)^c^				
	50,000 to <75,000 (n=1044)	412	34.6 (2.4)			13.6 (1.8)		11.1 (1.4)		8.2 (1.5)				
	≥75,000 (n=2443)	1111^c^	43.2 (1.9)^c^			22.6 (1.5)^c^		10.9 (1.2)		8.7 (0.8)^c^				
**Marital status**					.01									.20
	Married (n=3126)	1350^d^	41.7 (1.2)^c^			19.6 (1.1)^c^		12.0 (0.8)		8.7 (0.8)^c^				
	Unmarried (n=2920)	1167	36.2 (1.8)			15.5 (1.5)		11.2 (0.8)		8.1 (0.9)				
**Employment status**					.06									<.001
	Employed (n=2901)	1189	37.5 (1.5)			18.2 (1.2)^c^		9.3 (0.7)		8.8 (0.8)^c^				
	Unemployed (n=3145)	1328^c^	41.4 (1.4)^c^			17.4 (1.1)		14.5 (1.0)^c^		8.0 (0.7)				
**Rurality of residence**					.46									.12
	Metropolitan (n=5223)	2254^c^	39.9 (1.0)^c^			17.6 (0.8)		12.2 (0.7)^c^		8.7 (0.6)^c^				
	Micropolitan (n=470)	152	34.4 (4.6)			16.9 (4.1)		9.4 (1.9)		6.2 (1.5)				
	Small town (n=230)	80^c^	40.6 (9.0)^c^			26.7 (10.2)^c^		6.2 (2.1)		6.4 (2.3)				
	Rural (n=123)	31	31.4 (9.3)			12.8 (3.7)		7.2 (2.3)		10.1 (6.8)^c^				
**Health insurance**					<.001									<.001
	Yes (n=5542)	2374^c^	41.5 (1.1)^c^			19.0 (0.8)^c^		11.8 (0.7)^c^		9.1 (0.6)^c^				
	No (n=504)	143	21.2 (3.1)			7.8 (1.4)		10.3 (2.2)		2.4 (0.5)				
**Number of office visits per year**					<.001									<.001
	None (n=683)	109	11.9 (1.8)			4.3 (1.2)		5.9 (1.3)		1.3 (0.5)				
	1-4 (n=3832)	1530	38.7 (1.4)			18.0 (1.1)^c^		12.0 (0.8)^c^		7.6 (0.8)				
	≥5 (n=1531)	878^d^	58.3 (1.9)^c^			25.8 (2.1)^c^		14.3 (1.3)^c^		15.3 (1.4)^c^				
**General health status**					<.001									<.001
	Excellent/good (n=4974)	2000	37.3 (1.1)			17.6 (0.9)		10.9 (0.7)		7.6 (0.6)				
	Fair/poor (n=1072)	517^c^	49.2 (2.4)^c^			18.8 (3.0)^c^		15.4 (1.4)^c^		12.2 (1.6)^c^				
**Chronic medical condition**														
	Diabetes (n=1296)	620^c^	46.0 (2.5)^c^	.005		17.7 (2.2)		15.6 (1.4)^c^		10.5 (1.5)^c^		.001		
	High blood pressure (n=2710)	1203^c^	43.3 (1.7)^c^	.02		16.7 (1.5)		14.2 (0.9)^c^		10.5 (1.0)^c^		.001		
	Heart disease (n=609)	306^c^	49.5 (3.3)^c^	.003		18.2 (2.5)^c^		19.3 (2.4)^c^		9.8 (2.1)^c^		.008		
	Lung disease (n=816)	432^c^	50.4 (2.5)^c^	<.001		17.6 (1.3)		16.5 (1.8)^c^		13.2 (2.0)^c^		.001		
	Depression (n=1587)	913^c^	56.7 (2.1)^c^	<.001		24.6 (1.8)^c^		15.4 (1.5)^c^		14.1 (1.3)^c^		<.001		
	History of cancer (n=911)	407^c^	47.8 (2.8)^c^	.006		20.5 (2.3)^c^		13.6 (1.9)^c^		12.1 (1.6)^c^		.049		

^a^“Any” included video only (n=1110), audio only (n=876), and both (n=531)*.*

^b^Not applicable.

^c^Telehealth adoption use was higher than the average.

### Telehealth Motivations, Satisfaction, Perceptions, and Purposes

**Figure 1:**
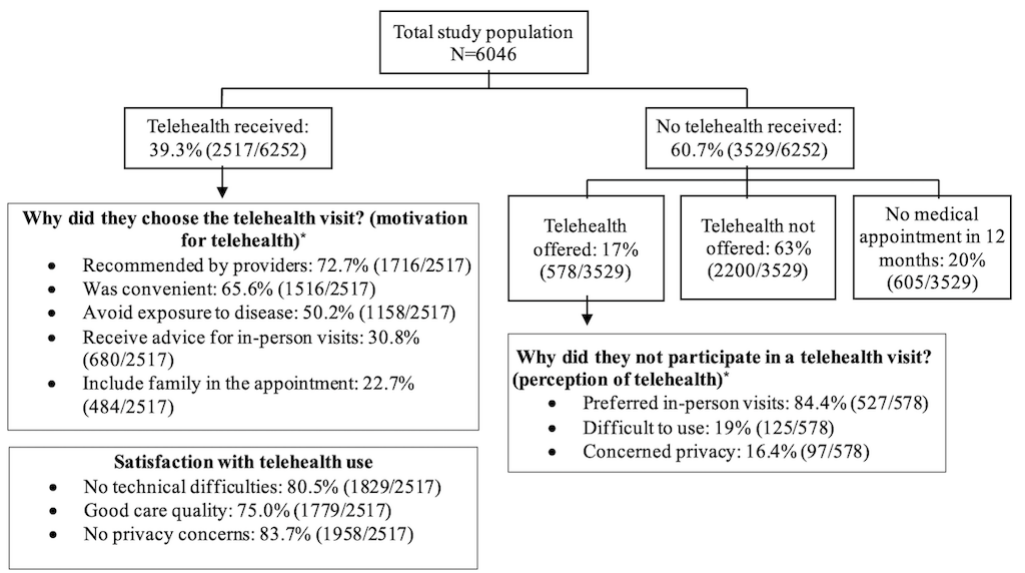
Study population flow chart (weighted %).

The primary reason for not using telehealth was the lack of an available telehealth option (2200/3529, 63%) or preference for in-person care if telehealth was offered (527/578, 84.4%; Figure 1). Provider's recommendation (1716/2517, 72.7%) and convenience (1516/2517, 65.6%) motivated people to use telehealth, and users were mostly satisfied with their telehealth visits, with no reported technical problems (1829/2517, 80.5%) or privacy concerns (1958/2517, 83.7%) and good care quality (1779/2517, 75%). Acute (600/2397, 29.7%) and chronic (583/2397, 21.4%) condition care were the most common purposes of telehealth use. However, the purpose of telehealth use differed by age (of those aged 18-34 years, 28.2% [99/381] used telehealth for mental health vs of those aged ≥75 years, 31.3% [93/272] used telehealth for chronic condition care), race/ethnicity (of non-Hispanic Asian individuals, 35.6% [41/124] used telehealth for acute care vs of individuals of other races/ethnicities, 29% [27/94] used telehealth for chronic condition care), and income (of those with an income <US $20,000, 27.5% [92/365] used telehealth for chronic condition care vs of those with an income ≥US $75,000, 34.9% [335/1085] used telehealth for acute care; Table 3 and Table S1 in Multimedia Appendix 2).

**Table 3:** Purpose for telehealth use by US adults in the third year of the COVID-19 pandemic (Health Information National Trends Survey [HINTS] 6, 2022).

Characteristics			Annual visit (n=485), weighted % (SE)		Acute minor illness (n=600), weighted % (SE)		Chronic medical condition management (n=583), weighted % (SE)		Medical emergency (n=46), weighted % (SE)		Mental health, behavioral, or substance abuse issues (n=337), weighted % (SE)		Other (n=346), weighted % (SE)
All adopters (n=2397)			17.8 (1.6)		29.7 (1.9)		21.4 (1.4)		1.6 (0.3)		15.7 (1.3)		13.8 (1.0)
**Age (years)**													
	18-34	19.5 (5.7)		28.0 (4.3)		9.3 (2.3)		2.3 (1.1)		28.2 (4.6)		12.7 (2.5)	
	35-49	11.3 (1.8)		36.5 (3.5)		19.6 (3.2)		2.0 (0.7)		17.1 (2.2)		13.5 (2.1)	
	50-64	17.0 (2.2)		30.0 (3.1)		27.9 (2.8)		0.4 (0.3)		11.2 (1.9)		13.5 (2.1)	
	65-74	26.2 (2.8)		22.6 (3.1)		29.4 (3.4)		1.2 (0.4)		6.4 (1.6)		14.2 (3.0)	
	≥75	28.6 (3.9)		17.4 (3.6)		31.3 (4.9)		2.6 (1.6)		0.8 (0.5)		19.3 (4.9)	
**Race/ethnicity**													
	Non-Hispanic White	16.1 (1.6)		32.5 (2.3)		19.6 (1.6)		1.1 (0.3)		17.6 (1.8)		13.1 (1.5)	
	Non-Hispanic Black/African American	27.4 (4.2)		23.9 (6.6)		25.9 (5.5)		2.0 (0.9)		6.8 (1.6)		14.0 (3.9)	
	Hispanic	13.9 (1.8)		28.0 (3.4)		22.3 (3.2)		3.5 (1.3)		13.1 (2.4)		19.2 (2.7)	
	Non-Hispanic Asian	18.8 (4.6)		35.6 (6.3)		22.4 (6.5)		1.6 (1.3)		14.8 (6.2)		6.8 (2.6)	
	Others	29.6 (19.4)		9.8 (4.4)		28.8 (11.1)		0.8 (0.7)		19.0 (7.6)		12.0 (5.2)	
**Household income (US $)**													
	<20,000	22.0 (3.6)		17.6 (4.8)		27.5 (5.7)		3.0 (1.2)		14.6 (3.1)		15.3 (3.3)	
	20,000 to <35,000	19.9 (3.4)		19.8 (6.8)		29.6 (5.0)		2.2 (1.0)		14.8 (3.2)		13.6 (4.5)	
	35,000 to <50,000	15.4 (3.2)		31.5 (5.4)		20.2 (3.2)		2.4 (1.9)		20.8 (7.8)		9.7 (1.8)	
	50,000 to <75,000	15.2 (3.1)		27.1 (4.1)		25.1 (4.2)		0.6 (0.5)		16.8 (3.3)		15.3 (2.5)	
	≥75,000	17.7 (2.6)		34.9 (2.9)		17.5 (1.8)		1.2 (0.4)		14.8 (1.7)		13.9 (1.4)	

### Factors Associated With Telehealth Use

Individuals who were younger (ORs 1.48-2.23, 18-64 vs ≥75 years), were female (OR 1.33, 95% CI 1.09-1.61), were Hispanic (OR 1.37, 95% CI 1.05-1.80; vs non-Hispanic Whites), or had at least a college education (OR 1.72, 95% CI 1.03-2.87; vs less than high school) had higher odds of using telehealth (Table 4). Unemployed (OR 1.25, 95% CI 1.02-1.54) or insured (OR 1.83, 95% CI 1.25-2.69) adults were more likely to use telehealth. Those who had 1 or more health care office visits in 1 year or had fair or poor general health had higher odds of using telehealth.

**Table 4:** Factors associated with telehealth use by 2517 US adults in the third year of the COVID-19 pandemic (Health Information National Trends Survey [HINTS] 6, 2022).

Factors			Results, adjusted OR^a,b^ (95% CI)		*P* value		Results, unadjusted OR (95% CI)		*P* value
**Age (years)**									
	18-34	1.59 (1.15-2.19)		.005		0.98 (0.74-1.29)		.87	
	35-49	2.23 (1.60-3.09)		<.001		1.45 (1.11-1.89)		.007	
	50-64	1.48 (1.10-1.99)		.01		1.13 (0.87-1.46)		.38	
	65-74	1.18 (0.86-1.63)		.23		1.06 (0.79-1.43)		.69	
	≥75	Reference		—^c^		Reference		—	
**Gender**									
	Female	1.33 (1.09-1.61)		.004		1.51 (1.26-1.80)		<.001	
	Male	Reference		—		Reference		—	
**Race/ethnicity**									
	Non-Hispanic White	Reference		—		Reference		—	
	Non-Hispanic Black/African American	0.90 (0.67-1.22)		.51		0.85 (0.65-1.11)		.24	
	Hispanic	1.37 (1.05-1.80)		.02		1.03 (0.82-1.29)		.82	
	Non-Hispanic Asian	1.08 (0.71-1.65)		.72		0.97 (0.61-1.55)		.91	
	Others	1.49 (0.88-2.54)		.14		1.52 (0.86-2.71)		.15	
**Education**									
	Less than high school	Reference		—		Reference		—	
	High school graduate	1.16 (0.70-1.92)		.56		1.93 (1.23-3.03)		.005	
	Some college	1.41 (0.84-2.37)		.19		1.19 (0.75-1.89)		.45	
	At least a college graduate	1.72 (1.03-2.87)		.04		1.52 (0.95-2.42)		.08	
**Household income (US $)**									
	<20,000	Reference		—		Reference		—	
	20,000 to <35,000	0.98 (0.70-1.38)		.90		1.00 (0.73-1.38)		.98	
	35,000 to <50,000	1.13 (0.74-1.72)		.57		1.08 (0.73-1.59)		.70	
	50,000 to <75,000	0.92 (0.67-1.26)		.60		0.94 (0.70-1.26)		.66	
	≥75,000	1.19 (0.85-1.67)		.32		1.34 (1.01-1.78)		.04	
**Employment status**									
	Employed	Reference		—		Reference		—	
	Unemployed	1.25 (1.02-1.54)		.03		1.18 (0.998-1.38)		.053	
**Marital status**									
	Married	1.15 (0.96-1.38)		.14		1.26 (1.05-1.52)		.02	
	Unmarried	Reference		—		Reference		—	
**Rurality of residence**									
	Metropolitan	Reference		—		Reference		—	
	Micropolitan	0.78 (0.55-1.11)		.17		0.79 (0.56-1.13)		.19	
	Small town	0.96 (0.56-1.64)		.88		0.69 (0.37-1.28)		.24	
	Rural	0.70 (0.33-1.47)		.34		1.03 (0.50-2.11)		.93	
**Health insurance**									
	Yes	1.83 (1.25-2.69)		.002		2.63 (1.84-3.76)		<.001	
	No	Reference		—		Reference		—	
**Number of visits**									
	None	Reference		—		Reference		—	
	1-4	4.33 (2.98-6.29)		<.001		4.69 (3.23-6.81)		<.001	
	≥5	9.26 (6.10-14.07)		<.001		10.37 (7.01-15.33)		<.001	
**General health status**									
	Excellent/good	Reference		—		Reference		—	
	Fair/poor	1.66 (1.30-2.13)		<.001		1.63 (1.27-2.09)		<.001	

^a^Adjusted for all variables in the table.

^b^OR: odds ratio.

^c^Not applicable.

## Discussion

### Principal Findings

Our study used nationally representative data to study modern telehealth practices observed in the third year of the COVID-19 pandemic. We comprehensively investigated features of telehealth use including adoption, motivation for use, purposes of use, respondent satisfaction, telehealth perceptions, and associated sociodemographic and health status characteristics of US survey participants. Approximately 2 in 5 adults received telehealth care, motivated primarily by providers’ recommendations and convenience. Care for both acute and chronic conditions was received via telehealth, with high overall patient satisfaction. Additionally, we identified factors associated with telehealth adoption, including younger age, female gender, Hispanic ethnicity, higher education status, being unemployed and insured, previously visiting a health care office, and poor health status. The wide array of descriptive findings and identified associations will help providers and health systems understand the factors that drive patients toward or away from telehealth visits. We illustrated the importance of the role of health policy to support those with low telehealth adoption and of integrating patients’ perspectives [24], as well as how telehealth adoption may improve inequities in health care access for socioeconomically marginalized groups [14].

In the United States, 39.3% of the population adopted telehealth during the third year of COVID-19. Video-only visits were more prevalent than audio-only visits, yet the mode used differed by sociodemographic characteristics. The main users of video visits were younger, educated, employed, insured, or high income–earning (≥US $75,000) adults, while the primary users of audio visits were older (≥75 years), of Hispanic ethnicity, unemployed, or low income–earning (<US $35,000), which is aligned with previous findings [8]. Given that video visits require technology-enabling environments (eg, broadband Internet access, digital device) and health technology literacy, our findings highlight the existence of inequities in video visit access. Although video and audio visits could serve different care needs, it is apparent that efforts will be necessary to improve the accessibility of video visits for disadvantaged groups.

In this study, the biggest barrier to telehealth adoption was a lack of accessibility despite its recent popularity. Although telehealth users' satisfaction was generally high, preference for in-person visits was still the main reason for not scheduling an available telehealth service. Interestingly, the ability of telehealth visits to include family members in patient visits was a major motivation for its utilization, highlighting the potential role of telehealth in improving the active engagement of patients and caregivers in clinical care, which could improve disease management [25]. Moreover, approximately one-third of participants mentioned that they used telehealth as a means of advice for in-person visits. This suggests that telehealth could help patients make informed health decisions and foster self-care practices, as telehealth could create an enabling environment for individuals to educate themselves using quality guidance for self-care [26]. As a preliminary visit, telehealth could reduce unnecessary office visits, potentially contributing to relieving health professionals’ burden and enhancing health care efficiency in addition to saving patients’ time and effort required to make a visit [27]. Further analyses on the cost-effectiveness of telehealth are suggested to examine the economic and efficiency impact of telehealth on the health care system.

Although acute minor illness care (eg, fever, sinus infection) was the most common purpose of a telehealth visit, telehealth use for chronic condition management (eg, diabetes, high blood pressure) was also high. The findings reveal that the scope of telehealth currently goes beyond acute care and is broadly used throughout multiple practice settings, which aligns with previous findings [28]. We observed the drivers behind telehealth use differed by age, race/ethnicity, and income. Notably, the youngest subgroup used telehealth mainly for mental health care. Non-Hispanic Black/African Americans, other ethnic groups, those with the lowest income, and older adults used televisits mainly for chronic conditions, while non-Hispanic White individuals, non-Hispanic Asian individuals, and those with the highest income used it for acute minor illnesses. The findings contribute to our understanding of how telehealth can improve different care needs for different subpopulations. Need-based targeted promotions could be considered to meet the observed care needs, including telepsychiatry for younger individuals or teleendocrinology and telecardiology for those with a low income or of older age. As the technology matures, we expect the features, implementation, and delivery of telehealth to vary based on specialty, care setting, and acuity. Our findings suggest that future development of telehealth tools may vary depending on the practice setting, including its use for behavior modification [29,30].

Younger individuals (18-64 years) were more likely to use telehealth than the oldest individuals (≥75 years), which aligns with the findings of a recent meta-analysis on telehealth use in cancer care [31]. Previously, older individuals had lower odds of using tablets and smartphones to communicate with providers [32]. Older adults’ low health technology literacy or hearing issues were identified as potential inhibiting factors for the use of health technology, including telehealth [33-35]. In this study, older adults (≥65 years) had higher prevalences of preferring in-person visits and difficulty using telehealth as reasons not to schedule available telehealth visits than their younger counterparts. Our findings suggest that targeted patient education, particularly in the context of health technology literacy or tailored care, could ensure smooth care delivery [35,36].

Women had 30% higher odds of using telehealth than men, which is aligned with existing evidence that women are more likely to make primary care visits [37]. However, during the early phase of the pandemic (March 2020-May 2021), men were nearly twice as likely to use telehealth than women in a surgical oncology center [9]. A notable observation from our study was that the reasons for telehealth visits differed by gender; there was a higher prevalence of telehealth use for mental health care among women (19% vs 12% for men; *P*=.03). Given that women had higher odds of poor mental health during the pandemic [38], we may need to further examine if telehealth could be a promising tool to address women’s mental health care needs.

Hispanic individuals were more likely to use telehealth than non-Hispanic White individuals. In contrast, during the early pandemic, Hispanic individuals were less likely to use telehealth than in-person office visits [10], and Hispanic cancer survivors were less likely to use email or the Internet to communicate with health providers compared with White cancer survivors [32]. One possible reason for the discrepancy in the findings from previous studies may be that Hispanic individuals were more impacted by COVID-19 infection than individuals of other race/ethnic groups during the early pandemic [39,40], which might have led them to using more hospital visits and less telehealth [10]. Another potential scenario for the high likelihood of telehealth use among Hispanic individuals could be that they may have health issues that could be appropriately handled through telehealth visits. Given that Hispanic individuals had a higher prevalence of other reasons (19%) for recent telehealth visits than non-Hispanic White or Black individuals (13%), further study is warranted to assess if there were unmet health care needs that could be categorized as other reasons in the Hispanic population and if telehealth can sustainably satisfy those [41].

Highly educated individuals (at least a college graduate) had increased odds of using telehealth than the least educated individuals (less than high school). Similarly, a lower education level was a known predictor of low adoption of health technology [42] and technology-based patient-provider communications during the early pandemic [32,43]. Although it is possible that other factors could be at play in this association, it is also possible that less-educated individuals’ concerns about the privacy of telehealth visits might have limited active telehealth use, as we witnessed in this study. Additional steps to ensure telehealth users’ privacy is protected may need to be taken, with continued efforts to secure health information safety.

Insured individuals had 80% higher odds of using telehealth than uninsured counterparts. Given that the major private health plans and Centers for Medicare and Medicaid Services started to cover telehealth since early in the pandemic [3], this is not surprising. Previously, an association was found between a lack of health insurance and low adoption of tablets or smartphones to communicate with providers [32]. Further discussions may need to be initiated on how to support and help uninsured individuals’ low telehealth use. Moreover, unemployed individuals were more likely to use telehealth than employed counterparts. Although there is limited evidence pertaining to this association in the literature, perhaps one interpretation could be that unemployed individuals had more health issues for which they could receive care through telehealth. We observed that unemployed individuals had poor health conditions and used telehealth visits for chronic condition care compared with their employed counterparts. On the other hand, it was notable that employed adults used telehealth primarily for acute minor illnesses than unemployed adults. Although further study is warranted to examine the purpose of telehealth visits by employment status to better understand the dynamics, our findings suggest that a tailored approach to enhance working individuals’ telehealth access and providing more telehealth options outside of regular office hours could be considered.

Individuals who had health care office visits at least once were significantly more likely to use telehealth than those without any office visits yearly, which aligned with the findings of a previous study that examined the relationship between frequent office visits and communicating through electronic health records with providers [32]. This association could also be interpreted as having more health issues and a poorer health condition. For example, individuals with frequent office visits (≥5 times a year) had higher prevalences of poor general health and of using telehealth for chronic condition care compared with those without health office visits. Our findings indicate that active telehealth users were those who were frequent in-person office visitors, rather than those who had been away from health care. Future assessments may need to focus on whether telehealth could accommodate individuals with long-term, consistent care needs as that could potentially reduce the burden on health care professionals particularly in the areas suffering from shortages of the health care labor force.

### Limitations

This study had some limitations. First, the temporality and causality of associations cannot be confirmed due to its cross-sectional design. Second, selection bias could be possible given the low response rate of 28.1%. However, HINTS is considered a high-quality national survey, full sample weights were applied to be representative, and imputation was conducted to minimize the bias from nonresponse. Third, as the HINTS is a self-report survey, reporting bias could also be present (eg, general health status is subjective). Fourth, although our data reflect telehealth use in the middle of the pandemic (data collected from March 2022 to November 2022), there may be changes in telehealth adoption and the factors associated with low adoption during the postemergency pandemic era. Hence, we suggest future studies to assess if there are any additional changes during the postemergency pandemic era starting in later 2023 and onward. Fifth, we were not able to look at asynchronous messaging as a mechanism of telecare and teledelivery due to data unavailability. Given its substantial use in practice, future studies are warranted to assess its trends and use [44]. Last, we did not consider health technology–related factors, including a technology-enabling environment (eg, digital device ownership, Internet connectivity) and health technology literacy, which are likely associated with telehealth use [45]. We would suggest further investigations into multiple factors at play for telehealth adoption in the future.

### Conclusions

Our findings from a nationally representative study of modern telehealth practices show that nearly 2 of 5 individuals in the United States used telehealth, motivated by providers’ recommendations and mainly for acute and chronic condition care, resulting in a positive experience. We identified that an age between 18 years and 64 years, female gender, Hispanic ethnicity, higher education, being unemployed, being insured, frequent health office visits, and poor health status were associated with telehealth use. Future research is warranted to assess the inhibiting factors for those with low telehealth adoption especially where telehealth could be an alternate for in-person care. Furthermore, we recommend evaluating if telehealth satisfies the care needs of those with several health comorbidities. These findings help providers and health systems understand the factors that drive patients to or away from telehealth visits as the technology becomes more routinely available across the United States beyond the needs of the pandemic, providing future directions for telehealth use and telehealth research.

## Appendix

Multimedia Appendix 1 Telehealth questionnaires.

Multimedia Appendix 2 Care purpose of recent telehealth visits by sociodemographic characteristics.

## Abbreviations

HINTS: Health Information National Trends Survey
OR: odds ratio
STROBE: Strengthening the Reporting of Observational Studies in Epidemiology

## Data Availability

The data for this study are publicly available at the National Cancer Institute website through the standard data access procedure [46].

## References

[1] Why use telehealth?. Health Resources & Services Administration.

[2] Hollander JE, Carr B (2020). Virtually perfect? Telemedicine for Covid-19. N Engl J Med.

[3] Billing for telebehavioral health. Health Resources & Services Administration.

[4] Notification of Enforcement Discretion for Telehealth Remote Communications During the COVID-19 Nationwide Public Health Emergency. US Department of Health and Human Services.

[5] Esposito S, Voccia E, Cantarelli A, Canali A, Principi N, Prati A, Parma COVID-19 Pediatric Working Group (PaCoPed) (2020). Telemedicine for management of paediatric infectious diseases during COVID-19 outbreak. J Clin Virol.

[6] Shokri T, Lighthall J (2020). Telemedicine in the era of the COVID-19 pandemic: implications in facial plastic surgery. Facial Plast Surg Aesthet Med.

[7] Bokolo AJ (2021). Application of telemedicine and eHealth technology for clinical services in response to COVID‑19 pandemic. Health Technol (Berl).

[8] Hare A, Adusumalli S, Mehrotra A, Bressman E (2022). Association between patient demographic characteristics and devices used to access telehealth visits in a US primary care network. JAMA Health Forum.

[9] Paro A, Rice D, Hyer J, Palmer E, Ejaz A, Shaikh C, Pawlik TM (2022). Telehealth utilization among surgical oncology patients at a large academic cancer center. Ann Surg Oncol.

[10] Weber E, Miller S, Astha V, Janevic T, Benn E (2020). Characteristics of telehealth users in NYC for COVID-related care during the coronavirus pandemic. J Am Med Inform Assoc.

[11] Ekwegh T, Cobb S, Adinkrah E, Vargas R, Kibe L, Sanchez H, Waller J, Ameli H, Bazargan M (2023). Factors associated with telehealth utilization among older African Americans in South Los Angeles during the COVID-19 pandemic. Int J Environ Res Public Health.

[12] Hung M, Ocampo M, Raymond B, Mohajeri A, Lipsky M (2023). Telemedicine among adults living in America during the COVID-19 pandemic. Int J Environ Res Public Health.

[13] Lee H, Singh G (2023). The impact of telemedicine parity requirements on telehealth utilization in the United States during the COVID-19 pandemic. J Public Health Manag Pract.

[14] Koenig LR, Becker A, Ko J, Upadhyay U (2023). The role of telehealth in promoting equitable abortion access in the United States: spatial analysis. JMIR Public Health Surveill.

[15] Yeung AWK, Torkamani A, Butte A, Glicksberg B, Schuller B, Rodriguez B, Ting DSW, Bates D, Schaden E, Peng H, Willschke H, van der Laak J, Car J, Rahimi K, Celi LA, Banach M, Kletecka-Pulker M, Kimberger O, Eils R, Islam SMS, Wong ST, Wong TY, Gao W, Brunak S, Atanasov AG (2023). The promise of digital healthcare technologies. Front Public Health.

[16] Survey Instruments. National Cancer Institute Health Information National Trends Survey.

[17] Westat (2020). Health Information National Trends Survey 5 (HINTS 5) Cycle 4 Methodology Report 2020. National Cancer Institute.

[18] von Elm E, Altman DG, Egger M, Pocock SJ, Gøtzsche PC, Vandenbroucke JP, STROBE Initiative (2007). The Strengthening the Reporting of Observational Studies in Epidemiology (STROBE) statement: guidelines for reporting observational studies. Ann Intern Med.

[19] Social Determinants of Health. Healthy People 2030.

[20] Methodology Reports. National Institute of Cancer Health Information National Trends Survey.

[21] Rural-Urban Commuting Area Codes. US Department of Agriculture Economic Research Service.

[22] Ioannidis JPA (2019). The importance of predefined rules and prespecified statistical analyses: do not abandon significance. JAMA.

[23] Di Leo G, Sardanelli F (2020). Statistical significance: *P* value, 0.05 threshold, and applications to radiomics-reasons for a conservative approach. Eur Radiol Exp.

[24] Michel J, Kawonga M, Rubin H (2023). Editorial: Pandemic-driven telehealth uptake: the missing healthcare provider, system and patient voices. Front Digit Health.

[25] Santana S, Lausen B, Bujnowska-Fedak M, Chronaki C, Kummervold P, Rasmussen J, Sorensen T (2010). Online communication between doctors and patients in Europe: status and perspectives. J Med Internet Res.

[26] Girault A, Ferrua M, Lalloué B, Sicotte C, Fourcade A, Yatim F, Hébert G, Di Palma M, Minvielle E (2015). Internet-based technologies to improve cancer care coordination: current use and attitudes among cancer patients. Eur J Cancer.

[27] Langabeer J, Champagne-Langabeer T, Alqusairi D, Kim J, Jackson A, Persse D, Gonzalez M (2016). Cost–benefit analysis of telehealth in pre-hospital care. J Telemed Telecare.

[28] Kuan PX, Chan W, Fern Ying Dk, Rahman M, Peariasamy K, Lai N, Mills Nl, Anand A (2022). Efficacy of telemedicine for the management of cardiovascular disease: a systematic review and meta-analysis. The Lancet Digital Health.

[29] Champion KE, Parmenter B, McGowan C, Spring B, Wafford Q, Gardner L, Thornton L, McBride N, Barrett El, Teesson M, Newton NC, Chapman C, Slade T, Sunderland M, Bauer J, Allsop S, Hides L, Stapinksi L, Birrell L, Mewton L (2019). Effectiveness of school-based eHealth interventions to prevent multiple lifestyle risk behaviours among adolescents: a systematic review and meta-analysis. The Lancet Digital Health.

[30] Gunasekeran DV, Tham Y, Ting DSW, Tan GSW, Wong TY (2021). Digital health during COVID-19: lessons from operationalising new models of care in ophthalmology. The Lancet Digital Health.

[31] Shaffer KM, Turner K, Siwik C, Gonzalez B, Upasani R, Glazer JV, Ferguson RJ, Joshua C, Low CA (2023). Digital health and telehealth in cancer care: a scoping review of reviews. The Lancet Digital Health.

[32] Kim J, Linos E, Fishman D, Dove M, Hoch J, Keegan T (2023). Factors associated with online patient-provider communications among cancer survivors in the United States during COVID-19: cross-sectional study. JMIR Cancer.

[33] Arcury TA, Sandberg J, Melius K, Quandt S, Leng X, Latulipe C, Miller DP, Smith DA, Bertoni AG (2020). Older adult Internet use and eHealth literacy. J Appl Gerontol.

[34] Norman CD, Skinner H (2006). eHealth literacy: essential skills for consumer health in a networked world. J Med Internet Res.

[35] The Lancet Haematology (2021). Telehealth and digital equity for older people. The Lancet Haematology.

[36] Sieck CJ, Sheon A, Ancker J, Castek J, Callahan B, Siefer A (2021). Digital inclusion as a social determinant of health. NPJ Digit Med.

[37] Hunt K, Adamson J, Hewitt C, Nazareth I (2011). Do women consult more than men? A review of gender and consultation for back pain and headache. J Health Serv Res Policy.

[38] Witteveen AB, Young IS, Cuijpers P, Ayuso-Mateos JL, Barbui I, Bertolini I, Cabello M, Cadorin C, Downes N, Franzoi D, Gasior M, Gray B, Melchior M, van Ommeren M, Palantza C, Purgato M, van der Waerden J, Wang S, Sijbrandij M (2023). COVID-19 and common mental health symptoms in the early phase of the pandemic: An umbrella review of the evidence. PLoS Med.

[39] Acosta AM, Garg S, Pham H, Whitaker M, Anglin O, O'Halloran A, Milucky J, Patel K, Taylor C, Wortham J, Chai SJ, Kirley PD, Alden NB, Kawasaki B, Meek J, Yousey-Hindes K, Anderson EJ, Openo KP, Weigel A, Monroe ML, Ryan P, Reeg L, Kohrman A, Lynfield R, Bye E, Torres S, Salazar-Sanchez Y, Muse A, Barney G, Bennett NM, Bushey S, Billing L, Shiltz E, Sutton M, Abdullah N, Talbot HK, Schaffner W, Ortega J, Price A, Fry AM, Hall A, Kim L, Havers FP (2021). Racial and ethnic disparities in rates of COVID-19-associated hospitalization, intensive care unit admission, and in-hospital death in the United States from March 2020 to February 2021. JAMA Netw Open.

[40] Nanchal R, Patel D, Guddati AK, Sakhuja A, Meersman M, Dalton D, Kumar G (2022). Outcomes of Covid 19 patients-Are Hispanics at greater risk?. J Med Virol.

[41] Silva MA, Perez O, Añez L, Paris M (2021). Telehealth treatment engagement with Latinx populations during the COVID-19 pandemic. The Lancet Psychiatry.

[42] Kontos E, Blake K, Chou W, Prestin A (2014). Predictors of eHealth usage: insights on the digital divide from the Health Information National Trends Survey 2012. J Med Internet Res.

[43] Zeng B, Rivadeneira N, Wen A, Sarkar U, Khoong E (2022). The impact of the COVID-19 pandemic on Internet use and the use of digital health tools: secondary analysis of the 2020 Health Information National Trends Survey. J Med Internet Res.

[44] Stephens J, Greenberg G (2022). Asynchronous telehealth. Prim Care.

[45] Cheng J, Arora V, Kappel N, Vollbrecht H, Meltzer D, Press V (2023). Assessing disparities in video-telehealth use and eHealth literacy among hospitalized patients: cross-sectional observational study. JMIR Form Res.

[46] Terms of Use. National Cancer Institute Health Information National Trends Survey.

