# Supplementary material for: Telehealth Utilization and Associations in the United States During the Third Year of the COVID-19 Pandemic: Population-Based Survey Study in 2022
Source: JMIR Public Health Surveill. 2024 Apr 26;10:e51279. doi: 10.2196/51279 (PMC11087857; doi:10.2196/51279)
Supplement: Multimedia Appendix 1 [file publichealth_v10i1e51279_app1.docx]

Supplemental file. Full survey questionnaires for telehealth from the Health Information National Trends Survey (HINTS 6, 2022)

D 1. A telehealth visit is a telephone or video appointment with a doctor or health professional.

In the past 12 months, did you receive care from a doctor or health professional using telehealth?

1. Yes, by video --> GO TO D4
2. Yes, by phone call (voice only with no video) --> GO TO D4
3. Yes, some by video and some by phone call --> GO TO D4
4. No telehealth visits in the past 12 months --> GO TO D2

D 2. In the past 12 months, were you offered the option to have a telehealth visit for any medical care you tried to schedule?

1. Yes --> GO TO D3
2. No --> **GO TO E1 on the next page**
3. I did not try to schedule any medical care in the past 12 months --> **GO TO E1 on the next page**

D 3. Did you choose **not** to participate in a telehealth visit for any of the following reasons?

a. I preferred to have the appointment(s) in person.....................................Yes [ ] / No [ ]

b. I was concerned about the privacy of telehealth visits............................Yes [ ] / No [ ]

c. I thought the telehealth technology would be difficult to use.................Yes [ ] / No [ ]

D 4. Why did you choose a telehealth visit(s) for yourself?

a. The health care provider recommended or required the visit use telehealth.

Yes [ ] / No [ ]

b. I wanted advice about whether I needed in-person medical care.

Yes [ ] / No [ ]

c. I wanted to avoid possible infection at the doctor’s office or hospital (e.g., COVID-19 or flu).

Yes [ ] / No [ ]

d. It was more convenient than going to the doctor (for example, less travel or wait times).

Yes [ ] / No [ ]

e. I could include family or other caregivers in my appointment.

Yes [ ] / No [ ]

D 5. What was the primary reason for your most recent telehealth visit?

**Mark only *one*.**

1. Annual visit
2. Minor illness/acute care (for example, fever, sinus infection)
3. Managing my chronic health condition/disease (for example, high blood pressure, diabetes, heart disease, obesity, cancer)
4. Medical emergency
5. Mental health, behavioral, or substance abuse issues (for example, depression, anxiety, drug or alcohol abuse)
6. Other

D 6. In general, how much do you agree or disagree with the following statements regarding your telehealth visit(s)?

1. a. I had technical problems with my telehealth visit(s) (for example, difficulty using the technology, trouble seeing or hearing my health care provider).
2. Strongly agree [ ]
3. Somewhat agree [ ]
4. Somewhat disagree [ ]
5. Strongly disagree [ ]
6. b. The care I received through telehealth was as good as a regular in-person visit.
7. Strongly agree [ ]
8. Somewhat agree [ ]
9. Somewhat disagree [ ]
10. Strongly disagree [ ]
11. c. I was concerned about the privacy of my telehealth visit(s).
12. Strongly agree [ ]
13. Somewhat agree [ ]
14. Somewhat disagree [ ]
15. Strongly disagree [ ]
