# Supplementary material for: Telehealth Utilization and Associations in the United States During the Third Year of the COVID-19 Pandemic: Population-Based Survey Study in 2022
Source: JMIR Public Health Surveill. 2024 Apr 26;10:e51279. doi: 10.2196/51279 (PMC11087857; doi:10.2196/51279)
Supplement: Multimedia Appendix 2 [file publichealth_v10i1e51279_app2.docx]

**Supplemental Table 1. Care purpose of recent telehealth visits**

**by sociodemographic characteristics**

|  | **Annual exam** | **Acute**  **minor illness^a^** | **Chronic medical condition^a^** | **Medical emergency** | **Mental health^a^** | **Other**  **reasons** | ***P*-value** |
| --- | --- | --- | --- | --- | --- | --- | --- |
|  | Weighted % (SE) | | | | | |  |
| **Average %^b^** | 17.8 (1.6) | 29.7 (1.9) | 21.4 (1.4) | 1.6 (0.3) | 15.7 (1.3) | 13.8 (1.0) |  |
| **Age** |  |  |  |  |  |  | <.0001 |
| 18-34 | 19.5 (5.7) | 28.0 (4.3) | 9.3 (2.3) | 2.3 (1.1) | 28.2 (4.6) | 12.7 (2.5) |  |
| 35-49 | 11.3 (1.8) | 36.5 (3.5) | 19.6 (3.2) | 2.0 (0.7) | 17.1 (2.2) | 13.5 (2.1) |  |
| 50-64 | 17.0 (2.2) | 30.0 (3.1) | 27.9 (2.8) | 0.4 (0.3) | 11.2 (1.9) | 13.5 (2.1) |  |
| 65-74 | 26.2 (2.8) | 22.6 (3.1) | 29.4 (3.4) | 1.2 (0.4) | 6.4 (1.6) | 14.2 (3.0) |  |
| $\geq$75 | 28.6 (3.9) | 17.4 (3.6) | 31.3 (4.9) | 2.6 (1.6) | 0.8 (0.5) | 19.3 (4.9) |  |
| **Gender** |  |  |  |  |  |  | .025 |
| Female | 14.4 (1.3) | 29.2 (1.9) | 22.1 (2.0) | 1.6 (0.4) | 18.5 (1.3) | 14.2 (1.4) |  |
| Male | 22.4 (3.1) | 30.2 (3.2) | 20.5 (2.1) | 1.6 (0.6) | 12.0 (2.6) | 13.3 (1.6) |  |
| **Race/Ethnicity^c^** |  |  |  |  |  |  | <.001 |
| NH White | 16.1 (1.6) | 32.5 (2.3) | 19.6 (1.6) | 1.1 (0.3) | 17.6 (1.8) | 13.1 (1.5) |  |
| NH Black/  African American | 27.4 (4.2) | 23.9 (6.6) | 25.9 (5.5) | 2.0 (0.9) | 6.8 (1.6) | 14.0 (3.9) |  |
| Hispanic | 13.9 (1.8) | 28.0 (3.4) | 22.3 (3.2) | 3.5 (1.3) | 13.1 (2.4) | 19.2 (2.7) |  |
| NH Asian | 18.8 (4.6) | 35.6 (6.3) | 22.4 (6.5) | 1.6 (1.3) | 14.8 (6.2) | 6.8 (2.6) |  |
| Other | 29.6 (19.4) | 9.8 (4.4) | 28.8 (11.1) | 0.8 (0.7) | 19.0 (7.6) | 12.0 (5.2) |  |
| **Household income** |  |  |  |  |  |  | .0005 |
| < $20,000 | 22.0 (3.6) | 17.6 (4.8) | 27.5 (5.7) | 3.0 (1.2) | 14.6 (3.1) | 15.3 (3.3) |  |
| $20,000 to < $35,000 | 19.9 (3.4) | 19.8 (6.8) | 29.6 (5.0) | 2.2 (1.0) | 14.8 (3.2) | 13.6 (4.5) |  |
| $35,000 to < $50,000 | 15.4 (3.2) | 31.5 (5.4) | 20.2 (3.2) | 2.4 (1.9) | 20.8 (7.8) | 9.7 (1.8) |  |
| $50,000 to < $75,000 | 15.2 (3.1) | 27.1 (4.1) | 25.1 (4.2) | 0.6 (0.5) | 16.8 (3.3) | 15.3 (2.5) |  |
| $\geq$ $75,000 | 17.7 (2.6) | 34.9 (2.9) | 17.5 (1.8) | 1.2 (0.4) | 14.8 (1.7) | 13.9 (1.4) |  |
| **Employment** |  |  |  |  |  |  | .0005 |
| Employed | 13.6 (1.5) | 35.7 (2.5) | 18.2 (1.6) | 1.9 (0.5) | 16.4 (2.1) | 14.2 (1.6) |  |
| Unemployed | 22.5 (2.6) | 22.8 (2.4) | 25.1 (2.3) | 1.2 (0.3) | 15.1 (1.7) | 13.3 (1.6) |  |
| **Number of office visits** |  |  |  |  |  |  | <.0001 |
| None | 36.9 (6.9) | 35.6 (8.5) | 9.3 (3.5) | 2.0 (2.1) | 3.8 (1.4) | 12.4 (5.3) |  |
| 1-4 times | 20.9 (2.2) | 32.0 (2.4) | 17.7 (1.9) | 1.5 (0.3) | 14.6 (2.0) | 13.3 (1.3) |  |
| $\geq$ 5 times | 9.1 (1.2) | 24.2 (2.60 | 30.3 (2.7) | 1.6 (0.7) | 19.6 (2.0) | 15.2 (1.9) |  |

a. Annual visit (N=485), acute minor illness (e.g., fever, sinus infection, N=600), chronic medical condition (e.g., high blood pressure, diabetes, heart disease, obesity, cancer, N=583), mental health, behavioral or substance abuse issues (e.g., depression, anxiety, drug or alcohol abuse, N=337), others (N=346), medical emergency (N=46); b. Weighted % was presented with SE, total N=2,397; c. NH: Non-Hispanic
